# Patterns and Levels of Sedentary Behavior and Physical Activity in a General Japanese Population: The Hisayama Study

**DOI:** 10.2188/jea.JE20170012

**Published:** 2018-05-05

**Authors:** Tao Chen, Hiro Kishimoto, Takanori Honda, Jun Hata, Daigo Yoshida, Naoko Mukai, Mao Shibata, Toshiharu Ninomiya, Shuzo Kumagai

**Affiliations:** 1 Faculty of Arts and Science, Kyushu University, Kasuga, Fukuoka, Japan; 2 Department of Epidemiology and Public Health, Graduate School of Medical Sciences, Kyushu University, Fukuoka, Japan; 3 Department of Behavior and Health Sciences, Graduate School of Human-Environment Studies, Kyushu University, Kasuga, Fukuoka, Japan

**Keywords:** epidemiology, sedentary time, physical activity, accelerometer, pattern of activity

## Abstract

**Background:**

The purpose of this cross-sectional study was to describe the patterns and levels of sedentary time and physical activity (PA) in a general Japanese population.

**Methods:**

A total of 1,740 community-dwelling Japanese adults aged ≥40 years participated in this study. Sedentary time and PA were assessed for 7 consecutive days using a tri-axial accelerometer. Daily patterns and levels of sedentary time and PA were calculated by sex, age group (40–64, 65–74, and ≥75 years), and body mass index (BMI; <25 and ≥25 kg/m^2^).

**Results:**

Participants spent half of their waking time being sedentary, 32.7% of which was accumulated in prolonged bouts ≥30 minutes, versus only 54.4 minutes/day (7% of waking time) as moderate-to-vigorous PA (MVPA) (11.8 minutes/day in bouts ≥10 minutes). In addition to total sedentary time, men had longer prolonged sedentary bouts and fewer breaks per sedentary hour than women. Similar trends were observed in participants aged ≥75 years and those with a higher BMI (≥25 kg/m^2^) compared to those with a younger age and lower BMI. Moreover, participants aged ≥75 years and those with a higher BMI accumulated fewer MVPA minutes in bouts ≥10 minutes. Only 34.8% of the population met the recommended level of ≥150 minutes/week MVPA in bouts ≥10 minutes.

**Conclusion:**

Japanese adults accumulated a large proportion of total sedentary time in prolonged bouts but few minutes in sustained bouts of MVPA, and few of them met the current PA guideline.

## INTRODUCTION

The health benefits of moderate-to-vigorous intensity physical activity (MVPA) throughout the life course have been well documented.^1^ Recently, emerging evidence has suggested that prolonged time in sedentary behavior, such as sitting or lying down with an energy expenditure ≤1.5 metabolic equivalent units (METs), increases the risk of cardiovascular disease, type 2 diabetes, and mortality independent of MVPA levels.^2^ Therefore, public health strategies aimed at increasing physical activity (PA) and reducing sedentary time in daily life are needed to improve and maintain health at the population level.

To implement effective intervention strategies to increase PA and reduce sedentary time, it is important to understand the usual patterns and levels of these behaviors. The use of an accelerometer allows the objective and accurate assessment of PA and sedentary time in population-based studies and reduces many sources of potential bias inherent to questionnaire-based measures.^3^ However, to date, daily PA or sedentary time in accelerometer-based studies has typically been summarized as the total amount of time spent in these behaviors. In addition to the total time, how PA and sedentary time are accumulated may also have health implications. For example, current PA guidelines indicate that MVPA should be accrued in sustained bouts ≥10 minutes for potential health benefits,^1^ although more research is needed to determine whether MVPA accumulated sporadically (in <10 consecutive minutes) has similar health impacts. Moreover, recent experimental studies demonstrated that uninterrupted sedentary time exerted detrimental cardiometabolic effects,^4^^,^^5^ and data from epidemiologic studies have also shown that sedentary time in shorter bouts (eg, more breaks in sedentary time) is associated with a favorable cardiometabolic profile.^6^

Despite the importance of patterns of PA and sedentary time, few accelerometer studies have simultaneously described how PA and sedentary time accumulate among adults.^7^^,^^8^ A recent study from Swedish CArdioPulmonary bioImage Study reported that only 29% of total MVPA time was accumulated in prolonged bouts (≥10 minutes), while over one-third of sedentary time was accumulated in longer bouts (≥20 minutes).^7^ However, these existing data are limited to Western populations. Thus, the generalizability of findings to countries with different environments, cultures, and lifestyles, such as Asian countries, is unclear. The purpose of this study was to describe patterns and levels of sedentary time and PA in a general Japanese population using a tri-axial accelerometer.

## METHODS

### Participants

The Hisayama Study, a population-based observational study of cardiovascular disease and its risk factors, started in 1961 in the Hisayama, a suburban town of Fukuoka metropolitan area in Southern Japan.^9^ In 2009, among 2,247 residents aged ≥40 years who participated in the health examination, a total of 1,987 (response rate: 88.4%) agreed to wear an accelerometer. Of them, 1,740 participants (87.5% of the accelerometer sample) with valid accelerometer data were included in the present study.

This study was approved by the Kyushu University Institutional Review Board for Clinical Research, and written informed consent was obtained from all participants.

### Sedentary behavior and physical activity measures

Sedentary time and PA were objectively measured using a tri-axial accelerometer (Active style Pro HJA-350IT; Omron Healthcare, Kyoto, Japan). Participants were ask to wear the accelerometer on either side of their waist during waking time for 7 consecutive days after the health examination and remove it for any water activities.

Data were recorded in 1-minute epoch. The accuracy of the intensity estimated by the Active style Pro has been validated with the Douglas bag method.^10^ The SAS macro program provided by the National Cancer Institute was used to compute non-wear time, with modifications based on our accelerometer.^11^ Non-wear time was defined as consecutive minutes of no activity (ie, estimated activity intensity <1.0 METs) for at least 60 minutes, allowing for 2 minutes of activities where intensity rose up to 1.0 METs.^12^^,^^13^ Data for participants with ≥10 hours of wear time per day for at least 4 wear days.^14^

The cut points used to define time spent in sedentary behavior and PA were as follows: ≤1.5 METs for sedentary time, 1.6–2.9 METs for light PA (LPA), 3.0–5.9 METs for moderate PA, and ≥6 METs for vigorous PA. Time spent in MVPA and vigorous PA were also calculated when PA accumulated in sustained bouts ≥10 minutes each with an allowance for up to 2 minutes below threshold.^15^ A prolonged sedentary bout was defined as ≥30 consecutive minutes of activity intensity ≤1.5 METs (a definition which has been reported to elicit detrimental cardiometabolic effects in an experimental-based study^5^). A sedentary break was defined as at least 1 minutes where the intensity of activity increased to and exceeded 1.5 METs following a sedentary bout.^16^

### Other measures

Body mass (kg) and height (m) were measured in light clothing and without shoes using standard protocols. Body mass index (BMI) was calculated as weight in kilograms divided by height in meters squared and then categorized into <25 and ≥25 kg/m^2^ groups. Because of small differences in sedentary time and PA across the 40–64-year age range based on a pilot examination, age was categorized into 40–64, 65–74, and ≥75 years groups.

### Statistical analysis

All statistical analyses were conducted using SAS version 9.3 (SAS Institute Inc., Cary, NC, USA). A significance level was set at two-sided α = 0.05. Time spent in a defined intensity was summed over valid days, and daily averages were then calculated. Daily averages of prolonged sedentary bouts were computed as: (1) total number of prolonged sedentary bouts; (2) total daily sedentary time accumulated in prolonged bouts; and (3) percentage of sedentary time accumulated in prolonged bouts ([sedentary time accumulated in prolonged bouts/total sedentary time] × 100). The daily average of sedentary breaks was computed as breaks per sedentary hour. The percentage of the study population meeting the current World Health Organization PA guideline was also analyzed according to the following criterion: accumulating 150 minutes per week MVPA or 75 minutes per week of vigorous intensity PA in bouts ≥10 minutes.^1^ Minutes per week of MVPA or vigorous PA was calculated as the average daily minutes of the valid wear days multiplied by 7. The accumulated weekly MVPA in bouts ≥10 minutes among those not meeting PA guideline were categorized into four groups (<37, 37 to <75, 75 to <113, and 113 to <150).^17^ The proportions of participants in these categories were also calculated and presented by sex, age and BMI groups.

The distributions of the majority of the activity variables were skewed; thus, the descriptive data are presented as median and interquartile range (IQR). Spearman’s correlation coefficients were calculated to examine associations between sedentary and PA variables. The Mann–Whitney U-test was used to test differences between sexes and BMI groups for each of the PA and sedentary variables. Differences between age groups for PA and sedentary variables were examined by the Kruskal-Wallis test, followed by the Dwass, Steel, and Critchlow-Fligner multiple pairwise comparison procedure to control the familywise error rate. Pearson’s chi-square analysis was used to identify differences between sex, age, and BMI groups in the proportion of participants meeting the current PA guideline.

## RESULTS

The median age of the 1,740 participants was 64 (IQR, 56–72) years, and 40.1% of the participants were men. The median accelerometer wear time was 14.1 (IQR, 13.0–15.4) hours/day.

Overall, participants spent 422.5 minutes (50% of wearing time) being sedentary, 363.4 minutes (43%) in LPA, and 54.4 minutes (7%) in MVPA daily. Sedentary time was negatively correlated with LPA (Spearman’s ρ = −0.56, *P* < 0.0001) and MVPA (Spearman’s ρ = −0.42, *P* < 0.0001), whereas LPA and MVPA were positively correlated (Spearman’s ρ = 0.34, *P* < 0.0001). As shown in Table 1, sedentary time was significantly longer in men, the oldest age group, and participants with a higher BMI than in women, the two younger age groups, and those with a lower BMI. Time spent in LPA and MVPA followed the opposite pattern for the sex, age, and BMI groups, showing significantly lower LPA and MVPA levels in men as well as across increasing strata for age and BMI.

**Table 1.** Volume of sedentary time and physical activity by sex, age, and BMI groups

	Sedentary time (min/day)	LPA (min/day)	MVPA (min/day)
All (*n* = 1,740)	422.5 (351.5–501.5)	363.4 (295.5–429.2)	54.4 (32.8–83.6)
Sex			
Men (*n* = 698)	448.9 (376.3–534.5)	311.8 (249.3–378.4)	50.0 (28.9–78.5)
Women (*n* = 1,042)	410.1 (341.9–474.4)^a^	391.6 (334.5–454.0)^a^	57.7 (37.1–88.5)^a^
Age groups			
40–64 years (*n* = 899)	416.4 (350.3–491.0)	380.8 (316.9–439.3)	61.7 (42.4–91.7)
65–74 years (*n* = 540)	419.1 (343.6–490.3)	356.1 (287.1–422.6)^b^	55.7 (34.5–86.0)^b^
≥75 years (*n* = 301)	454.1 (376.9–556.9)^b,c^	323.5 (254.5–390.5)^b,c^	27.2 (11.3–53.0)^b,c^
BMI groups			
<25 kg/m^2^ (*n* = 1,323)	419.1 (346.3–491.8)	368.2 (299.7–432.6)	56.3 (34.4–87.6)
≥25 kg/m^2^ (*n* = 417)	446.1 (364.2–529.4)^d^	350.4 (280.9–413.0)^d^	49.8 (29.7–73.4)^d^

BMI, body mass index; LPA, light physical activity; MVPA, moderate-to-vigorous physical activity.

Values are median (interquartile range).

^a^Significant sex difference (*P* < 0.05) vs men.

^b^Significant age group difference (*P* < 0.05) vs 40–64 years;

^c^Significant age group difference (*P* < 0.05) vs 65–74 years;

^d^Significant BMI group difference (*P* < 0.05) vs ≥25 kg/m^2^.

The entire study participants had 3.0 prolonged bouts (≥30 minutes) of sedentary time per day and spent 133.6 minutes (32.7% of the total sedentary time) in prolonged bouts (Table 2). Time in prolonged sedentary bouts was highly correlated with total sedentary time (Spearman’s ρ = 0.79, *P* < 0.0001). Moreover, sedentary time was broken 9.2 times per sedentary hour. Men had greater numbers of prolonged sedentary bouts, longer prolonged bouts, and had fewer breaks per sedentary hour compared to women. Similar trends were observed in older participants and those with a higher BMI compared to those with younger age and a lower BMI.

**Table 2.** Patterns of tri-axial accelerometer-measured sedentary time by sex, age, and BMI

	Number of prolonged sedentary bout^a^ (times/day)	Time spent in prolonged sedentary bout^a^ (min/day)	Percentage of total sedentary time in prolonged sedentary bout^a^ (%)	Number of breaks^b^ per sedentary hour (times/hour)
All (*n* = 1,740)	3.0 (2.2–4.0)	133.6 (89.5–199.3)	32.7 (23.7–43.1)	9.2 (7.2–11.2)
Sex				
Men (*n* = 698)	3.2 (2.4–4.6)	149.2 (98.3–236.7)	35.8 (25.7–46.8)	8.5 (6.4–11.0)
Women (*n* = 1,042)	2.8 (2.0–3.7)^c^	121.7 (81.8–178.0)^c^	31.3 (22.8–40.6)^c^	9.4 (7.8–11.4)^c^
Age groups				
40–64 years (*n* = 899)	2.8 (2.0–3.6)	123.1 (81.1–175.0)	29.9 (22.1–39.5)	10.0 (8.2–12.0)
65–74 years (*n* = 540)	3.0 (2.3–4.1)^d^	139.6 (93.3–202.7)^d^	34.3 (25.0–44.1)^d^	8.6 (6.8–10.7)^d^
≥75 years (*n* = 301)	3.6 (2.4–5.1)^d,e^	179.0 (110.6–279.1)^d,e^	39.8 (28.3–51.7)^d,e^	7.3 (5.8–9.3)^d,e^
BMI groups				
<25 kg/m^2^ (*n* = 1,323)	2.9 (2.1–3.9)	129.1 (86.0–192.2)	32.1 (23.3–42.4)	9.3 (7.3–11.3)
≥25 kg/m^2^ (*n* = 417)	3.1 (2.4–4.3)^f^	148.3 (97.6–217.0)^f^	35.0 (25.7–45.5)^f^	8.8 (6.8–10.8)^f^

BMI, body mass index.

Values are median (interquartile range).

^a^A prolonged sedentary bout was defined as ≥30 min of time in continuous sedentary time where activity intensity ≤1.5 METs, with no allowance of interruption.

^b^A sedentary break was defined as at least 1 minute where the intensity of activity rose up to and above 1.5 METs following a sedentary bout.

^c^Significant sex difference (*P* < 0.05) vs men.

^d^Significant age group difference (*P* < 0.05) vs 40–64 years;

^e^Significant age group difference (*P* < 0.05) vs 65–74 years;

^f^Significant BMI group difference (*P* < 0.05) vs ≥25 kg/m^2^.

The median daily MVPA in prolonged bouts ≥10 minutes was 11.8 minutes/day (Table 3). There was no sex difference in MVPA minutes accumulated in bouts ≥10 minutes. Time spent in MVPA in bouts ≥10 minutes was significantly lower in the oldest age group compared to the two younger age groups. Time spent in MVPA in bouts ≥10 minutes was also significantly lower in those with a higher BMI compared to those with a lower BMI.

**Table 3.** Patterns of tri-axial accelerometer-measured MVPA and prevalence of participants meeting the physical activity guideline by sex, age, and BMI

	MVPA in bouts ≥10 min (min/day)	≥150 min/week MVPA in bouts of ≥10 min, %
All (*n* = 1,740)	11.8 (2.4–31.5)	34.8
Sex		
Men (*n* = 698)	14.3 (2.4–34.4)	37.8
Women (*n* = 1,042)	11.0 (2.6–29.7)	32.7^a^
Age groups		
40–64 years (*n* = 899)	12.7 (3.9–33.0)	35.9
65–74 years (*n* = 540)	15.0 (3.1–35.7)	41.1
≥75 years (*n* = 301)	3.6 (0–15.7)^b,c^	19.9^b,c^
BMI groups		
<25 kg/m^2^ (*n* = 1,323)	12.6 (2.8–33.0)	36.7
≥25 kg/m^2^ (*n* = 417)	9.0 (1.7–25.9)^d^	28.5^d^

BMI, body mass index; MVPA, moderate-to-vigorous physical activity.

Values are median (interquartile range).

^a^Significant sex difference (*P* < 0.05) vs men.

^b^Significant age group difference (*P* < 0.05) vs 40–64 years;

^c^Significant age group difference (*P* < 0.05) vs 65–74 years;

^d^Significant BMI group difference (*P* < 0.05) vs ≥25 kg/m^2^.

**Figure 1.**
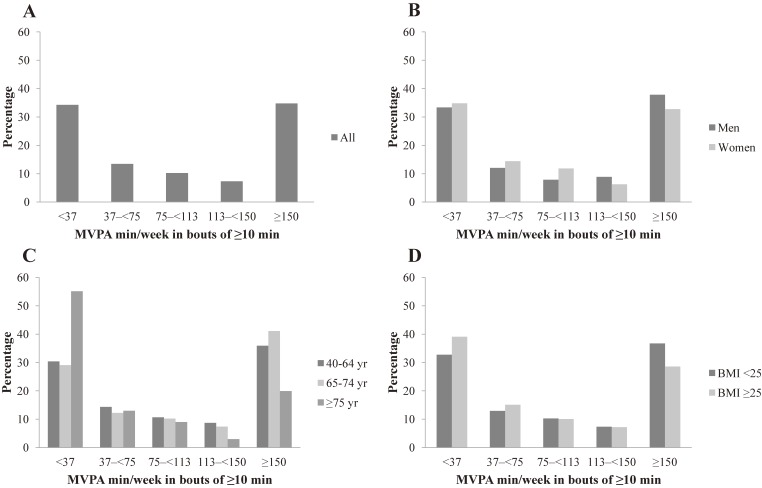
Distribution of participants accumulated defined weekly MVPA in bouts of ≥10 min by sex, age, and BMI. BMI, body mass index; MVPA, moderate-to-vigorous physical activity.

A total of 34.8% of the participants adhered to the PA guideline, defined as ≥150 minutes/week MVPA, in bouts ≥10 minutes (Table 3). A larger proportion of men than women adhered to the PA guideline. Participants in the oldest age group and those with a higher BMI had a significant lower adherence to the PA guideline than the two younger age groups and those with a lower BMI. Figure 1 (A, B, C, and D) shows the proportion of participants accumulating defined levels of weekly MVPA in bouts ≥10 minutes among all participants and by sex, age, and BMI groups. Except for the group aged ≥75 years, the distribution of participants showed a similar pattern across subgroups (Figure 1). Over one-third (34.5%) of the participants were in the <37 minutes/week MVPA category, ranging from 33.5% to 35.2% between sex groups, 29.3% to 56.2% across age groups, and 33.0% to 39.6% between BMI groups.

## DISCUSSION

This study provides the first detailed description of patterns and levels of sedentary time and PA in a general Japanese population with regard to sex, age, and BMI. A main finding was that >7 hours of the day was spent being sedentary, more than one-third of which was accumulated in prolonged bouts ≥30 minutes. Men, those of older age, and those with a higher BMI spent more total time and had more numerous prolonged bouts of sedentary time compared to their counterparts. Regarding MVPA, participants spent only 54.4 minutes/day as MVPA (11.8 minutes/day when considering only minutes from bouts ≥10 minutes), and only 34.8% of the population accumulated the recommended level of ≥150 minutes per week of MVPA in bouts ≥10 minutes.

Overall, participants in this study spent 50% of accelerometer wear time being sedentary, 43% in LPA, and 7% in MVPA. Data from the Reasons for Geographic and Racial Differences in Stroke (REGARDS) study showed that white and black American adults aged ≥45 years spent 75–90% of their time be sedentary versus only 0–1.7% in MVPA.^17^ In the Swedish ABC (attitude, behavior, and change) study using a nationally representative sample, data showed that Swedish adults also spent more time being sedentary (57.1%) and less time in LPA (39.1%) and MVPA (3.8%).^18^ Given the evidence that a greater BMI is associated with physical inactivity and greater sedentary time,^19^^,^^20^ the differences may be partly attributed to BMI differences between the study samples, as 76.4% of 7,967 participants in the REGARDS study and 45% of 1,172 participants in the Swedish ABC study were classified as overweight and obese (BMI ≥25), while only 23.8% of the present participants fell into this category. Differences in other sample characteristics, including age, race, and environmental factors, or differences in device used may also partly account for the discrepant result. Thus, differences between studies should be interpreted cautiously.

The significant differences in each variable across the age and BMI groups observed in this study are in agreement with previous findings demonstrating that PA decrease and sedentary time increases with increasing age^17^^–^^19^ and BMI.^17^^,^^20^^,^^21^ Moreover, men spent more time being sedentary and less time in LPA than women, findings that are consistent with those of previous reports.^7^^,^^19^^,^^22^ Previous findings on the relationship between PA and sex are inconsistent. Observations based on Western populations indicate that men had more time in MVPA than women,^7^^,^^17^^,^^21^^,^^22^ while this sex difference was not observed in Chinese adults aged 40–75 years old.^23^ In the present study, men spent slightly less time in MVPA than women, which is in concordance with previous observations in a large sample of older Japanese men and women.^24^ Of several factors that may cause the inconsistent finding in sex differences, the use of different accelerometers and data processing procedures could be most influential. Most accelerometers used in previous studies are uni-axial, and many validation studies are based primarily on locomotive activities, which were found to significantly underestimate non-locomotive PA, such as many household activities.^25^ Considering gender roles, women have more involvement in household activities, the intensity of which would have been underestimated in previous studies.^25^ The tri-axial accelerometer used in the present study is able to estimate PA intensity more accurately by its PA classification algorithms for locomotive and non-locomotive activities compared to the conventional uni-axial accelerometer.^10^ Indeed, data from a previous study used the same device showed women spent more time in non-locomotive MVPA than men, although the difference was not significant for the ≥70 year age group.^26^ Thus, it is possible that the underestimated intensity of household activities might lead to underestimating MVPA in women analyzed in previous studies.

Over one-third of the total sedentary time was accumulated in prolonged, uninterrupted bouts (≥30 minutes), with 9.2 breaks per sedentary hour in the present study. Although directly comparing these studies would be somewhat problematic, the proportion of total sedentary time accumulated in prolonged bouts is comparable to what has been reported in previous studies. Among 7,247 middle-aged and older women in the Women’s Health Study, prolonged sedentary bouts ≥30 minutes accounted for 31.5% of total sedentary time,^27^ while among a national sample of 8,096 American men and women aged ≥45 years, prolonged sedentary bouts ≥30 minutes represented 48.0% of the total sedentary time.^28^ In line with previous reports,^7^^,^^28^ participants who were male, were older, and had a higher BMI tended to accumulate longer periods of prolonged sedentary time in addition to total sedentary time in the present study. Consistent with previous studies,^7^^,^^28^ men and individuals who were older with a higher BMI had fewer breaks per sedentary hour (also indicating more time spent in longer sedentary bouts). These findings, coupled with recent findings, suggest that the factors associated with total sedentary time may also influence patterns of accumulated sedentary time. Given the large proportion of prolonged sedentary time across subgroups observed in the present study and prolonged bouts of sedentary time shown to be particularly harmful,^5^^,^^6^ the implementation of interventions regarding sedentary time should be prioritized in the future.

Of concern, adults in the present study accumulated a mere 11.8 minutes/day of MVPA in prolonged bouts ≥10 minutes. Men tend to spend less time in total MVPA than women with levels reversing for MVPA bouts, although the difference was not significant. Consistent with previous studies, sustained MVPA bouts decrease with increasing BMI and advancing age.^17^^,^^19^^,^^22^ Notably, the most marked differences were observed for oldest age group, with 3.6 minutes of MVPA bouts in individuals aged ≥75 years compared to 12.7–15.0 minutes in individuals aged <75 years, which may be attributed to the changes in health status associated with aging. Corresponding with the measured MVPA in bouts, only 34.8% of Japanese participants met the current PA guideline of 150 minutes of weekly MVPA in sustained bouts ≥10 minutes. The prevalence is higher than those that have been reported in the United States,^29^^,^^30^ United Kingdom,^31^ and Canada^21^ defined as the same PA guideline criterion. As mentioned earlier, differences in population characteristics (eg, BMI) and devices may contribute to the discrepant results. Despite the prevalence of the population meeting PA guideline is commonly reported, less is known about the distribution of weekly MVPA among those not meeting PA guideline. A recent data from the REGARDS study showed that the distribution of participants exhibited a reverse J-shaped configuration across the weekly MVPA categories. The present study observed similar findings, and it is noteworthy that over one-third of the participants accumulated less than 37 minutes/week of sustained MVPA, which raises serious public health concerns given the harmful health consequences of physical inactivity. Considering a large proportion of participants lacking bouts of MVPA, interventions to increase sustained MVPA in the population are needed, although further studies are also needed to determine the health impact of PA considering bout duration.

The strengths of this study include the relatively large Japanese population-based design and the use of a tri-axial accelerometer to assess PA and sedentary time, allowing a more accurate estimate of activity intensity than a conventional uni-axial accelerometer.^10^ Some limitations, however, should be considered when interpreting our findings. First, the generalizability of the findings is somewhat limited because the present study was performed in a single Japanese town, so study with a nationally representative sample is needed in the future. Second, the recognized limitations of accelerometers include their inability to detect some types of PA (eg, swimming and cycling) and differentiate sitting and standing. Third, the excluded participants were younger than those included in the present study. It is possible that the participants in this study were less sedentary and more active than those who were excluded. Hence, sedentary time may be underestimated and PA level may be overestimated. Furthermore, information regarding sociodemographic factors (eg, education and occupation), status of disability, mental health issues, and environmental factors (eg, neighborhood walkability), which might be related to PA and sedentary time, was not available for the present analysis. The consideration of these factors is needed to fully understand sex-, BMI-, and aging-related differences in patterns and levels of PA and sedentary time.

### Conclusions

In conclusion, one-half of the waking time of this general Japanese population was spent being sedentary, more than one-third of which as prolonged, uninterrupted bouts of sedentary time. Men, those of older age, and those with a higher BMI tended to accumulate longer prolonged sedentary bouts and fewer breaks per sedentary hour in addition to total sedentary time than their counterparts. Moreover, Participants aged ≥75 years and those with a higher BMI accumulated fewer MVPA minutes in bouts ≥10 minutes. Overall, the participants spent only a few minutes in sustained bouts of MVPA regardless of sex, age, and BMI status, and only 34.8% of them met the current PA guideline. These data suggest that there is a great need for interventions to reduce prolonged sedentary bouts ≥30 minutes and promote MVPA in bouts ≥10 minutes among Japanese adults.
